# Electrophysiological and Behavioral Responses of *Phthonoloba viridifasciata* (Lepidoptera: Geometridae) to Volatiles from *Alsophila spinulosa*

**DOI:** 10.3390/insects17060554

**Published:** 2026-05-28

**Authors:** Qi Sun, Weicheng Yang, Sheng Liang, Mengqing Zhou, Fen Liu, Xianyu Li, Bibo Zhou, Tianyu Liang

**Affiliations:** 1 School of Life Sciences, Guizhou Normal University, Guiyang 550025, China; 242100100407@gznu.edu.cn (Q.S.); qixiaobao0427@163.com (M.Z.); 15117707714@163.com (F.L.); 19306513218@163.com (X.L.); 19306518595@163.com (B.Z.); 19985972222@163.com (T.L.); 2 Chishui Alsophila National Nature Reserve Management Bureau, Chishui 646259, China; 17785964995@163.com

**Keywords:** *Alsophila spinulosa*, plant volatile compounds, host location, dynamic headspace adsorption

## Abstract

*Phthonoloba viridifasciata* (Lepidoptera: Geometridae) is the primary herbivorous insect of the rare and endangered plant *Alsophila spinulosa*. The aim of this study was to investigate the host-seeking mechanism of *P. viridifasciata* by screening volatile compounds from *A. spinulosa* for their attractant or repellent activity, so as to provide a theoretical basis for its eco-friendly control. We used the dynamic headspace adsorption to collect the volatile compounds from *A. spinulosa*. A combination of Gas Chromatography–Electroantennography Detection (GC-EAD), Gas Chromatography–Mass Spectrometry (GC-MS), electroantennography (EAG), and Y-olfactometer bioassays was used to screen and identify active compounds that induce responses in *P. viridifasciata*. Five active volatile compounds induced significant electrophysiological activity, including decane, undecane, 4-ethylbenzaldehyde, m-ethylacetophenone, and p-ethylacetophenone. All five active compounds triggered EAG responses in both sexes. Behavioral bioassays further revealed that 4-ethylbenzaldehyde (10^−2^ and 10^−4^ mol/L) and decane (10^−5^ mol/L) act as female attractants. Meanwhile, males showed a behavioral preference response to 4-ethylbenzaldehyde at 10^−2^ mol/L.

## Simple Summary

*Alsophila spinulosa*, a rare ancient fern known as a “living fossil,” is seriously damaged by *Phthonoloba viridifasciata*. Plant volatiles play a crucial role in the host-seeking behavior of herbivorous insects. This study collected and analyzed the volatile compounds released by *A. spinulosa*, identifying five compounds that elicited electrophysiological responses in this moth. The results indicated that 4-ethylbenzaldehyde at 10^−2^ mol/L exhibited a significant attractant effect on both male and female moths. In subsequent experiments, this compound was used as the core active ingredient, providing a foundation for the development of attractants for pest control.

## 1. Introduction

*Alsophila spinulosa* (Cyatheaceae: Alsophila) is the only large arborescent fern in the world. Originating in the Carboniferous, it appeared before the dinosaurs and is therefore known as a “living fossil” of terrestrial plants [1,2]. *Phthonoloba viridifasciata* (Lepidoptera: Geometridae) is the primary herbivorous pest of the rare and endangered fern [3,4,5]. Female moths lay eggs on the tender leaves during their unfolding stage, and larvae feed on the leaves of *A. spinulosa*; during population outbreaks, entire leaves may be consumed, severely impacting the spore production of *A. spinulosa* [3,6]. Currently, control methods for *P. viridifasciata* are limited to basic physical and chemical control. The main measures include removing the withered stipes of *A. spinulosa* to destroy pupation sites, thereby reducing the eclosion rate, and applying a low dose of insecticide during the early larval stage [6]. Moreover, owing to the complex life history and high reproductive capacity of the pest, these conventional methods are difficult to achieve effective results in a short period, and no efficient control strategy has been established to date. Therefore, the development of eco-friendly control methods is crucial for sustainable management of *P. viridifasciata* and protection of *A. spinulosa*.

Insects primarily rely on plant volatile compounds (PVOCs) to accurately locate their hosts in complex environments [7,8,9]. PVOCs are low-molecular-weight, highly volatile substances released by plants during growth and development or in response to external stimuli, including hydrocarbons, alcohols, aldehydes, esters, and other oxygen-containing compounds [10,11,12]. Various organs of plants can emit volatile compounds, such as leaves, stems, and flowers [13,14,15]. Moreover, plants can release volatile compounds throughout plant developmental stages, particularly during key stages including leaf expansion, flowering, and fruit ripening [16,17,18]. VOCs not only facilitate plant communication but also mediate many interactions within plant–insect communities [19,20]. For herbivorous insects, plant volatiles provide important chemical cues for long-distance host recognition and localization, as well as facilitate the search for nutritional resources, oviposition sites, and mating partners [21,22,23]. For instance, *Phthorimaea operculella* (Lepidoptera: Gelechiidae) was significantly attracted to cis-3-hexen-1-ol, nonanal, and decanal from tobacco volatiles [24]. The Indian meal moth exhibited a significant difference in oviposition preference among normal-oleic and high-oleic peanuts [25]. These authors found that 4-ethylbenzaldehyde and 2,5-dimethylbenzaldehyde derived from high-oleic peanuts exerted a significant repellent effect on female moths [25]. Butyl hexanoate and cis-3-hexen-1-ol were identified as key host volatiles that stimulate oviposition by *Grapholita molesta* (Lepidoptera: Tortricidae) [26]. In addition to mediating host location by herbivores, plant volatiles can also attract the natural enemies of herbivorous insects to reduce plant damage [27,28]. For example, when corn was damaged by *Spodoptera frugiperda* (Lepidoptera: Noctuidae) larvae, it released linalool and β-caryophyllene, which attracted parasitic wasps of *Trichogramma* (Hymenoptera: Trichogrammatidae) to locate the eggs of *S. frugiperda* [29]. Similarly, *aloe* plants infested by *Crioceris asparagi* (Coleoptera: Chrysomelidae) larvae released increased levels of linalool and α-pinene, which attracted more parasitic wasps [30].

Based on this ecological principle, the development of insect attractants and repellents using plant-derived bioactive compounds has become a key research direction for environmentally friendly pest management. Compared with conventional chemical pesticides, plant-derived behavioral regulators have advantages such as abundant sources, easy biodegradability, and relative safety toward non-target organisms. Pest control methods utilizing PVOCs have been widely applied in various fields; for example, during periods of severe insect infestations in tomato crops, limonene and methyl salicylate significantly reduced *Aleyrodidae* populations, while limonene application increased tomato fruit yield by 32% [31]. Additionally, 1,2-diethylbenzene can be used to prepare attractants for controlling the diamondback moth (*Plutella xylostella*) [32]. Thus, systematic research on plant-derived insect behavioral regulators not only deepens the understanding of chemical interaction mechanisms between plants and insects, but also provides a theoretical basis and technical support for the development of efficient, eco-friendly pest control products.

In this context, we collected volatile compounds from *A. spinulosa* using the dynamic headspace adsorption method. Then, we used Gas Chromatography–Electroantennographic Detection and Gas Chromatography–Mass Spectrometry to identify and screen the active compounds that could induce electroantennographic responses in *P. viridifasciata*. Potential attractants and repellents were further selected through EAG and behavioral assays. This study aimed to reveal the behavioral mechanism of host location in *P. viridifasciata* and to provide a theoretical basis for pest control and monitoring.

## 2. Materials and Methods

### 2.1. Test Insects and Plants

Larvae of *P. viridifasciata* were collected from the *Alsophila* National Nature Reserve, Chishui City, Guizhou Province, China (105°58′ E, 28°27′ N). The collected larvae were brought back to the laboratory and reared in an artificial climate incubator (RGLC-P1500-B3, Hefei Daskat Biotechnology, Hefei, China) under controlled conditions: temperature 26 ± 1 °C and relative humidity 70 ± 5%. After emergence, female and male adults were reared separately in insect rearing cages. Adults aged 2–3 days post-eclosion, which were sexually mature, unmated, and possessed fully expanded wings, intact legs and antennae as well as vigorous activity, were used in this experiment.

For the plant materials, 2-year-old *A. spinulosa* seedlings were used in the experiment. The seedlings were planted in a 1:1:1 mixture of peat soil (5–20 mm, Pindstrup, Ryomgård, Denmark), perlite, and vermiculite and cultivated in greenhouse at Chishuii *Alsophila* National Nature Reserve. The growth conditions were maintained as follows: temperature 24 ± 2 °C, relative humidity 80 ± 2%, and photoperiod 14L: 10D (light: dark). Seedlings in good growth condition, without pest or disease damage and not exposed to chemical pesticides, were used in this study.

### 2.2. Collection of Volatiles from A. spinulosa

Volatile compounds from *A. spinulosa* were collected using the dynamic headspace adsorption method. The connection mode of all sampling parts was as per those described by Luo et al. [33]. Adsorption tubes were filled with 300 mg of PoraPak™ QS adsorbent (80–100 mesh, Waters, Milford, MA, USA). Adjacent devices were connected with PVC tubing, and all connections were sealed with Parafilm (Bemis Company, Inc., Neenah, WI, USA) to ensure the airtightness of the collection system. During sampling, purified air was pumped into the collection bag at an inlet flow rate of 0.6 L/min, and volatiles were trapped at an outlet flow rate of 0.4 L/min for a continuous 7 h period. Volatiles from each seedling were collected in three biological replicates at the same time, under controlled conditions: temperature 24 ± 2 °C and relative humidity 80 ± 2%, light intensity 3000 lux, with an empty collection bag (without seedlings) used as a control. After sampling, each adsorption tube was eluted with n-hexane (Shanghai Macklin Biochemical Co., Ltd., Shanghai, China) and stored at −20 °C for subsequent analysis.

### 2.3. GC-EAD Responses and Identification of Volatiles from A. spinulosa

Under a stereomicroscope (TS-80, Shanghai Yoke Instrument Co., Ltd., Shanghai, China), antennae were excised at the base using ophthalmic scissors, followed by cutting off a small segment at each antennal tip. The reference electrode of the PRG-3 probe was connected to the base of the antenna, and the recording electrode was connected to the antenna tip. The eluted crude extract was concentrated to 0.5 mL via nitrogen stream before GC-EAD analysis (7820A-IDAC 4, Agilent Technologies, Santa Clara, CA, USA; Syntech GmbH, Buchenbach, Germany). GC Separation was performed on an HP-5 column (30 m × 0.32 mm × 0.25 μm, Agilent Technologies); the injector temperature was 250 °C, and splitless injection was used. Column temperature program was as follows: initial temperature 40 °C for 2 min, increased to 100 °C at 5 °C/min and held for 1 min, then raised to 280 °C at 15 °C/min. Mass spectrometry conditions: electron impact ionization at 70 eV, ion source temperature 230 °C, mass scanning range 50–500 *m*/*z*. The EAD baseline was monitored in the GC-EAD software (GcEad-1.2.5., Syntech GmbH, Buchenbach, Germany) until stabilization. Subsequently, 3 μL of the concentrated volatile extract of *A. spinulosa* was injected for GC-EAD measurement.

The EAD-active compounds were identified by GC-MS. GC-MS (7820A-5975, Agilent Technologies, USA) operating conditions were identical to those of the GC-EAD, and mass spectra were matched with reference databases using MassHunter software (MassHunter GC/MS Acquisition 10.1.49). Volatile components of *A. spinulosa* were identified by comparing their retention times with authentic standards, and their relative contents were calculated via the peak area normalization method; the formula was used according to what was described by Ling et al. [34]. Candidate volatile compounds triggering antennal electrophysiological responses in *P. viridifasciata* were preliminarily identified by correlating EAD response curves with FID chromatographic peaks.

### 2.4. Antennae Response of P. viridifasciata to Volatile Compounds

GC-EAD technology can separate complex mixed volatiles via gas chromatography and specifically screen olfactory-active components for preliminary active compound identification. Subsequently, EAG is used to perform concentration gradient verification on the single active components identified by GC-EAD, quantify antennal response intensity to determine the optimal effective concentration, and eliminate false-positive responses [35,36]. Therefore, volatile compounds from *A. spinulosa* were prepared into five concentration gradients (10^−1^, 10^−2^, 10^−3^, 10^−4^, and 10^−5^ mol/L) using n-hexane as the solvent. A 10 μL of test solution was dropped onto a clean filter paper strip (10 mm × 30 mm), the flow rate of the purified continuous air stream was adjusted to 1.0 L/min, and the stimulation airflow to 0.2 L/min. Each stimulus lasted 0.5 s, with a 30 s interval between continuous stimuli to allow full recovery of the antennal sensory organs. During the assay, antennae were first stimulated with n-hexane. After this initial treatment, test compounds were applied in ascending order of concentration, with one stimulation per concentration; finally, antennae were stimulated again with n-hexane. The average value was used as the experimental result.

### 2.5. Behavioral Responses of P. viridifasciata to Active Substances

The behavioral sensitivity of male and female *P. viridifasciata* adults (at least 300 individuals) to different concentrations of *A. spinulosa* volatiles was tested using an Y-olfactometer according to the method of Wang et al. [25]. Before the assay, adults of *P. viridifasciata* were starved for at least 4 h in a clean, ventilated room without external odors. During the test, 30 μL of each volatile solution was dropped onto a filter paper, with an equal volume of n-hexane as the blank control; the two filter papers were placed in odor source bottles connected to the two arms of the Y-olfactometer, respectively, and the airflow of each arm was set to 0.4 L/min. Only one adult was introduced at a time for the assay, and its behavior was observed for 10 min. An insect was recorded as exhibiting a preference if it entered the first one-third region of an arm and remained there for more than 30 s; otherwise, it was scored as showing no choice. To reduce interference from background color, the Y-olfactometer was placed on white paper during all tests. The behavioral response results were calculated using the formula described by Ling et al. [34].

### 2.6. Data Statistical Analysis

The normality and homoscedasticity of all data were tested prior to analysis using Shapiro–Wilk and Levene test. The relative EAG response values of different volatile compounds were compared by two-way ANOVA, followed by Tukey’s HSD test. EAG response value among tested concentrations of each VOC were analyzed by repeated measures one-way ANOVA, followed by Tukey’s HSD test. Behavioral responses between different concentrations of active compounds and the n-hexane control were analyzed via the chi-square (*χ*^2^) test. All statistical analyses were performed using SPSS 27.0 (IBM SPSS Statistics 27.0, New York, NY, USA), and statistical graphs were generated using Origin 2025b.

## 3. Results

### 3.1. GC-EAD Responses of P. viridifasciata to Volatile Compounds from A. spinulosa

Volatile compounds from *A. spinulosa* collected by headspace dynamic adsorption were identified using GC-MS. A total of 22 volatiles were identified. The most abundant compound was p-ethylacetophenone (25.91%), followed by m-ethylacetophenone (16.42%) and dodecane (10.81%); nine compounds accounted for more than 3% of the total volatiles, whereas the relative contents of the remaining compounds were relatively low (Table A1).

**Figure 1:**
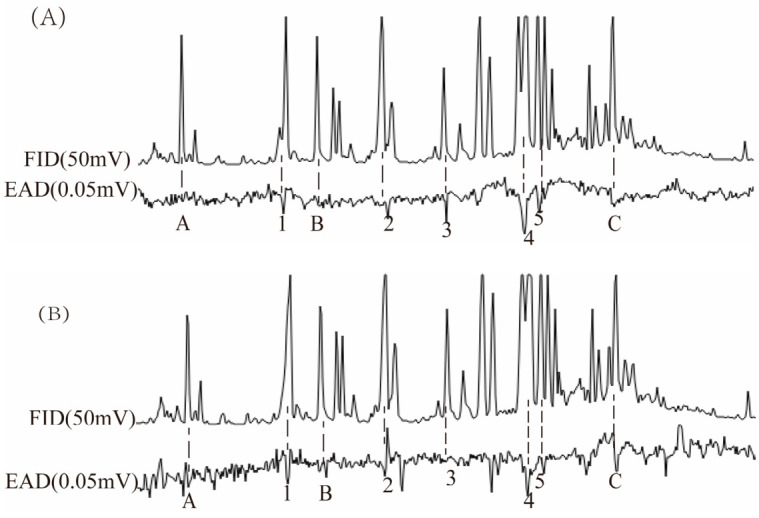
Responses of *Phthonoloba viridifasciata* to volatiles from *Alsophila spinulosa* as measured by Gas Chromatography–Electroantennographic Detection (GC-EAD). (**A**) Male. (**B**) Female. 1: decane, 2: undecane, 3: 4-ethylbenzaldehyde, 4: m-ethylacetophenone, 5: p-ethylacetophenone. All compounds represented by these letters are artificial compounds and are unrelated to the subsequent experimental results. A: n-butyl ether, B: 2-ethyl-1-hexanol, C: dimethyl phthala.

GC-EAD results showed that five volatile compounds from *A. spinulosa* elicited EAG responses in *P. viridifasciata* (Figure 1). These compounds were identified by GC-MS as decane, undecane, 4-ethylbenzaldehyde, m-ethylacetophenone, and p-ethylacetophenone.

### 3.2. Antennae Responses of P. viridifasciata to Different Volatiles from A. spinulosa

**Figure 2:**
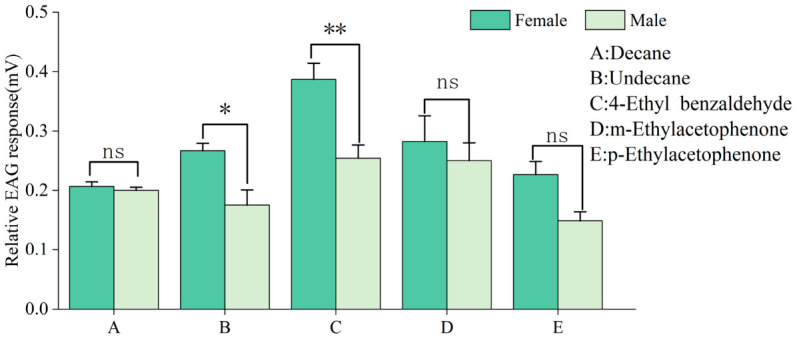
Electroantennogram (EAG) responses of *P. viridifasciata* to active compounds from *A. spinulosa*. Bars represent the relative EAG values of adults at 10^−1^ mol/L (mean ± SE). Asterisks indicate significant differences among different sexes according to two-way ANOVA followed by Tukey’s HSD test (* *p* < 0.05, ** *p* < 0.01, ns means no significant difference).

*P. viridifasciata* exhibited varying antennae responses to different volatiles at the same concentration (10^−1^ mol/L), with significant sex differences (Figure 2). All five compounds elicited EAG responses in both sexes.

Female adults exhibited stronger EAG responses to 4-ethylbenzaldehyde (EAG = 0.387 ± 0.027 mV) among all tested compounds, while showing the weakest EAG response to decane (EAG = 0.206 ± 0.008 mV). The relative EAG values of females to undecane were significantly higher than those of males (*p* < 0.05); additionally, the females’ EAG response to 4-ethylbenzaldehyde was remarkably higher than that of males (*p* < 0.01). No significant sexual differences were observed in the responses to decane, p-ethylacetophenone and m-ethylacetophenone.

### 3.3. EAG Responses of P. viridifasciata to Volatiles from A. spinulosa at Different Doses

**Figure 3:**
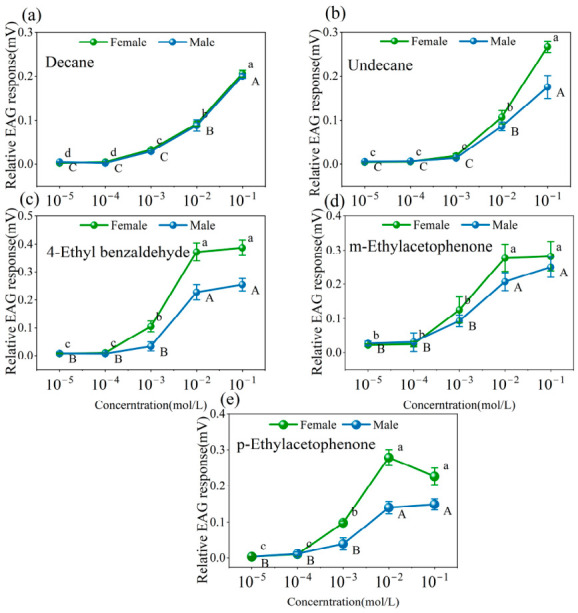
Relative electroantennogram responses (mean ± SE) of adult *P. viridifasciata* to volatile compounds at different concentrations (10^−1^, 10^−2^, 10^−3^, 10^−4^, and 10^−5^ mol/L). (**a**) Decane, (**b**) undecane, (**c**) 4-ethylbenzaldehyde, (**d**) m-ethylacetophenone, (**e**) p-ethylacetophenone. Different letters indicate significant differences (repeated measures one-way ANOVA, Tukey’s HSD test, *p* < 0.05).

Figure 3 shows the EAG responses of *P. viridifasciata* to five concentration gradients (10^−1^, 10^−2^, 10^−3^, 10^−4^, and 10^−5^ mol/L) of the synthetic standard volatile compounds. Undecane did not induce significant EAG responses in either sex at low concentrations (10^−3^, 10^−4^, and 10^−5^ mol/L). When the concentration reached 10^−2^ mol/L, both males and females exhibited significant EAG responses, with the response peaking at 10^−1^ mol/L; males showed significantly stronger EAG responses to decane at 10^−2^ mol/L than at lower concentrations, while female adults showed significant responses at 10^−3^ mol/L. 4-Ethylbenzaldehyde induced a significant EAG response in males at 10^−2^ mol/L, which did not differ significantly from that at the highest concentration. For females, significant EAG responses were observed starting at 10^−3^ mol/L, and the response plateaued at 10^−2^ mol/L. In contrast, m-ethylacetophenone induced a significant EAG response in both male and female adults at a concentration of 10^−2^ mol/L. The EAG response value of female adults to p-ethylacetophenone reached the maximum at 10^−2^ mol/L and decreased at 10^−1^ mol/L, while the EAG response value of male adults to 4-ethylacetophenone peaked at 10^−1^ mol/L.

### 3.4. Behavioral Responses of P. viridifasciata to A. spinulosa

**Figure 4:**
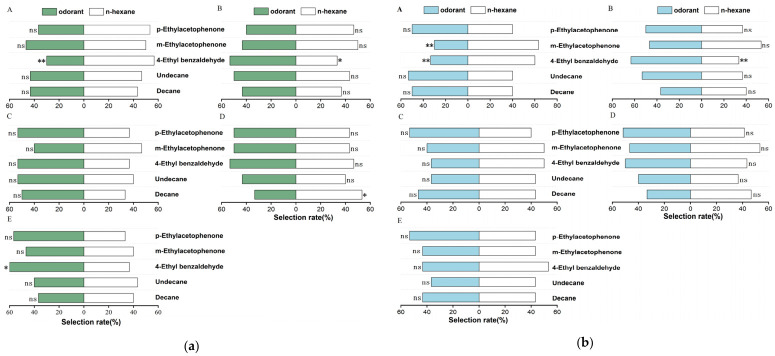
Behavioral responses of *P. viridifasciata* to active substances at different concentrations. (**a**) females, (**A**): 10^−1^ mol/L; (**B**): 10^−2^ mol/L; (**C**): 10^−3^ mol/L; (**D**): 10^−5^ mol/L; (**E**): 10^−4^ mol/L; (**b**) males, (**A**): 10^−1^ mol/L; (**B**): 10^−2^ mol/L; (**C**): 10^−3^ mol/L; (**D**): 10^−4^ mol/L; (**E**): 10^−5^ mol/L. Asterisks indicate significant differences between the experimental group and the control group (chi-square (*χ*^2^) test, * *p* < 0.05, ** *p* < 0.01, ns means no significant difference).

Male and female adults of *P. viridifasciata* exhibited differential choice responses to the five active volatiles from *A. spinulosa* at different concentrations (Figure 4). At 10^−1^ mol/L, both males and females exhibited strong repellent responses to 4-ethylbenzaldehyde (females *χ*^2^ = 8.379, *p* = 0.004, *p* < 0.01; males *χ*^2^ = 7.839, *p* = 0.005, *p* < 0.01). m-ethylacetophenone did not induce a significant choice response in females but showed a significant repellent effect in males (*χ*^2^ = 11.71, *p* = 0.001, *p* < 0.01); no obvious repellent or attractive responses were observed toward the remaining compounds. At 10^−2^ mol/L, 4-ethylbenzaldehyde showed strong attractive effects on both sexes (females *χ*^2^ = 4.651, *p* = 0.031, *p* < 0.05; males *χ*^2^ = 9.375, *p* = 0.002, *p* < 0.01). At 10^−3^ mol/L, the females and males showed no response to any of these compounds. At low concentrations (10^−4^ and 10^−5^ mol/L), males showed no obvious responses to any compounds at 10^−4^ and 10^−5^ mol/L; 4-ethylbenzaldehyde at 10^−4^ mol/L (*χ*^2^ = 5.454, *p* = 0.02, *p* < 0.05) had a significant attractive effect on females, while decane at 10^−5^ mol/L (*χ*^2^ = 4.651, *p* = 0.031, *p* < 0.05) exerted significant repellent effects on females.

## 4. Discussion

*A. spinulosa* has important scientific value in speciation and paleoclimate research. In addition, it has medicinal and ornamental value, and acts as the primary host plant of *P. viridifasciata*. This pest was first discovered in 2019 in the Chishui *Alsophila* Nature Reserve, Guizhou Province, China, increasing at a rate of five generations per year, posing a serious threat to the growth and reproduction of *A. spinulosa* [5,6]. Previous studies have demonstrated that volatile compounds emitted by *A. spinulosa* provide crucial chemical cues for *P. viridifasciata* for host location, feeding and oviposition, and play a crucial role in regulating its life activities [37,38]. Therefore, developing species-specific attractants and repellents based on these plant volatiles can offer new strategies for the monitoring, trapping, and behavioral regulation of *P. viridifasciata*. In this study, volatile compounds from *A. spinulosa* were collected using the dynamic headspace adsorption and analyzed by GC-MS; a total of 22 plant volatiles were identified. There is a low degree of similarity between our results and those reported by Xiao et al., which may be due to methodological differences [38]. Xiao et al. collected and identified volatiles from *A. spinulosa* using solid-phase microextraction (SPME) coupled with GC-MS [38]. In addition, numerous studies have demonstrated that plant volatiles are influenced by biotic factors, such as insect herbivory, pathogens, and symbiotic microorganisms [39,40,41], as well as abiotic factors including temperature, drought, and light [42,43,44], which may also be important factors underlying the differences in volatile composition observed among different studies. In addition, five active compounds from the volatiles of *A. spinulosa* that elicited EAG responses in *P. viridifasciata* were preliminarily screened and identified using GC-EAD and GC-MS, including decane, undecane, 4-ethylbenzaldehyde, m-ethylacetophenone, and p-ethylacetophenone. Notably, several artificial compounds were identified among the volatile compounds in the experiment. GC-EAD results showed that these compounds could elicit antennal responses in *P. viridifasciata* (refer to the letter-labeled peaks in Figure 1). Accordingly, it is essential to distinguish between endogenous components and exogenous contaminants in the experiment, which can effectively avoid the interference of contaminants on experimental results and ensure accurate and rational analysis of insect olfactory response mechanisms.

These substances were verified using EAG after preliminary screening by GC-EAD; the results showed that all these compounds could elicit electroantennogram responses in *P. viridifasciata*, though sensitivities to different compounds varied between sexes. Females exhibited significantly higher EAG responses than males to undecane, and extremely significantly higher responses to 4-ethylbenzaldehyde. Consistent with the results of this study, several previous studies have reported similar sexual differences in EAG responses; for example, female *Bactrocera dorsalis* (Diptera: Tephritidae) showed significantly higher EAG responses than males to five plum volatile compounds, including sorbitol ester, ethyl hexanoate, and hexyl isobutyrate [34]. Female *Graphium sarpedon* (Lepidoptera: Papilionidae) also showed stronger EAG responses to host volatiles compared to males [45]. Additionally, terpinene, bornyl acetate, and limonene from *Populus tomentosa* volatiles elicited higher EAG values in female *Bemisia tabaci* (Gennadius) (Hemiptera: Aleyrodidae) than in males [46]. In summary, female *P. viridifasciata* are more sensitive to *A. spinulosa* volatiles, which may be related to the fact that herbivorous insects exhibit sexual differences in the type and number of olfactory sensilla on their antennae [47,48,49]. Chen et al. further confirmed using scanning electron microscopy that female *P. viridifasciata* have significantly longer antennae than males, and the length of sensilla trichodea I, which is responsible for detecting volatile compounds, was also significantly greater in females [50]; these morphological differences provide a direct structural basis that female moths rely more on plant volatiles to host recognition, localization and oviposition site selection.

Significant responses of both sexes to undecane, and m-ethylacetophenone, as well as significant responses of males to p-ethylacetophenone, decane and 4-ethylbenzaldehyde, were all initiated at 10^−2^ mol/L. In contrast, significant responses of females to decane and 4-ethylbenzaldehyde, were initiated at 10^−3^ mol/L. The differing concentration thresholds for significant EAG responses among compounds and between sexes further demonstrate that the electrophysiological responses of *P. viridifasciata* to host volatiles exhibit obvious thresholds. Similar patterns have been reported in other insects. For instance, *Asias halodendri* showed relatively weak EAG responses to low concentrations of jujube volatiles; the response increased markedly at 10 μg/μL and peaked at 100 μg/μL [51]. *Athetis dissimilis* showed weak EAG responses to host volatiles at low concentrations (10^−4^, 10^−3^ mol/L), and electrophysiological activity was significantly enhanced with increasing concentration [52].

Compared with n-hexane, 4-ethylbenzaldehyde at concentrations of 10^−2^, 10^−3^, and 10^−4^ mol/L exhibited an attractant effect on adult females, with attraction rates of 53.33%, 53.33%, and 60.00%, indicating that this volatile compound plays a key regulatory role in the host-seeking behavior of *P. viridifasciata*. The higher sensitivity of females to plant volatiles is closely associated with their need to find suitable mating and oviposition sites, and thereby secure adequate living space and food sources for their offspring [22,53,54]. Plant volatiles can act as important complementary cues that enhance males’ ability to locate females [55]. This study also found that male insects showed significant positive orientation toward 4-ethylbenzaldehyde, indicating that this compound has potential attractiveness. Based on this finding, future research could combine plant volatiles with sex pheromones to develop more effective trapping or disruption systems, thereby enabling precise management of male insects.

In this study, undecane and p-ethylacetophenone elicited significant EAG responses in *P. viridifasciata* but induced no obvious behavioral effects among the five concentration gradients. These results indicate that there is no direct correlation between the EAG responses and behavioral selection results of *P. viridifasciata* to the active volatiles. This discrepancy may be partly due to the fact that the EAG technique only detects chemoreceptor activity on the antennae and thus merely reflects the sensitivity of antennal sensilla to volatile compounds [56,57]. Another reason is that insect responses to chemical stimuli involve complex processes. Insects first detect chemical signals in the environment via chemoreceptors, transduce them into electrical signals, and relay these signals to the central nervous system. After information integration by the central nervous system, the motor system is activated to trigger behavioral responses such as orientation, attraction, repellence, or feeding [58,59]. In addition to olfactory cues, insects behavioral responses to volatiles are also influenced by visual stimuli, physiological state, sexual maturity, and other factors [60,61,62]. For example, *Sitophilus zeamais* exhibited the strongest response to a combination of yellow color and maize volatile odor, which was far stronger than its response to color or odor alone [63]. Mated female *Manduca sexta* exhibited enhanced sensitivity and responsiveness to host plants [64].

In this study, only several volatile compounds were selected for EAG and behavioral bioassays, and their biologically effective concentrations and active components were identified. However, plant volatile compounds in natural conditions are very complex, and insects generally exhibit higher sensitivity to odor blends consisting of various compounds at specific proportions [65,66,67]. Therefore, future studies should evaluate the EAG and behavioral responses of *P*. *viridizonata* to additional volatile components from *A*. *spinulosa*, including compounds that did not elicit obvious GC-EAD responses. The most attractive or repellent compound combinations can be further screened and proportionally optimized to formulate odor blends with trapping effects comparable to those of *A. spinulosa* plants and to thoroughly elucidate the underlying behavioral regulatory mechanisms. On this basis, field trials should be performed to validate trapping efficacy, which will provide systematic, comprehensive, and reliable theoretical bases and technical support for the monitoring of population dynamics, eco-friendly control, and the development of environment-friendly plant-derived attractants and repellents against *P. viridifasciata*.

## Data Availability

The data supporting the results of this study are available from the corresponding author upon reasonable request.

## Appendix A

**Table A1:** Relative concentrations and components of plant volatiles from *Alsophila spinulosa*.

No.	Retention Time/min	Substance	Molecular Formula	CAS	Content/%
1	5.67	3-Hexanone	C_6_H_12_O	589-38-8	0.10%
2	9.40	Styrene	C_8_H_8_	100-42-5	0.08%
3	9.81	Nonane	C_9_H_20_	111-84-2	1.01%
4	10.66	Benzene, (1-methylethyl)-	C_9_H_12_	98-82-8	0.27%
5	10.98	alpha-Phellandrene	C_10_H_16_	99-83-2	0.06%
6	11.94	Benzaldehyde	C_7_H_6_O	100-52-7	0.04%
7	13.33	Butanoic acid, butyl ester	C_8_H_16_O_2_	109-21-7	1.38%
8	13.45	Decane	C_10_H_22_	124-18-5	7.12%
9	14.64	Butanedioic acid, dimethyl ester	C_6_H_10_O_4_	106-65-0	0.13%
10	14.48	2-Ethylhexan-1-ol	C_8_H_18_O	104-76-7	6.13%
11	15.63	Acetophenone	C_8_H_8_O	98-86-2	1.07%
12	16.59	Benzoic acid, methyl ester	C_8_H_8_O_2_	93-58-3	1.87%
13	16.79	Undecane	C_11_H_24_	1120-21-4	9.96%
14	16.92	Nonanal	C_9_H_18_O	124-19-6	0.75%
15	18.81	Benzaldehyde, 4-ethyl-	C_9_H_10_O	4748-78-1	5.02%
16	19.72	Dodecane	C_12_H_26_	112-40-3	10.81%
17	20.95	m-Ethylacetophenone	C_10_H_12_O	22,699-70-3	16.42%
18	21.26	p-Ethylacetophenone	C_10_H_12_O	937-30-4	25.91%
19	21.44	Tridecane	C_13_H_28_	629-50-5	7.53%
20	22.69	Tetradecane	C_14_H_30_	629-59-4	3.31%
21	22.98	2-Epi-alpha.-funebrene	C_15_H_24_	65,354-33-8	0.41%
22	23.07	cis-beta-Farnesene	C_15_H_24_	28,973-97-9	0.60%
